# 

**Published:** 1895-08

**Authors:** 


### CONTENTS FOR AUGUST, 1895.

| ORIGINAL COMMUNICATIONS. | PAGE. |
|---|---|
| Remarks on Cancer of the Breast. By HERMAN MYNTER, M. D. | 1 |
| Infantile Scurvy. By IRVING M. SNOW, M. D. | 4 |
| A Case of Spina Bifida Treated by Operation, Including Closure of the Spinal Canal by a Celluloid Plate. By ROSWELL PARK, M. D. | 8 |
| The Histological Conformation of the Medulla. By WILLIAM C. KRAUSS, M. D. | 12 |
| A Study in Heads. By JULIUS POHLMAN, M. D. | 17 |
| Ectopic Pregnancy. By HENRY D. INGRAHAM, M. D. | 18 |
| Two Cases of Ectopic Gestation—Retention of the Fetus for Eight and Thirteen Years Respectively—Operation—Cure. By MATTHEW D. MANN, A. M., M. D. | 23 |
| Backache from Weak Heart. By CHARLES G. STOCKTON, M. D. | 29 |
| Paroxysmal Sneezing. By W. SCOTT RENNER, M. D. | 31 |
| Report of a Case of Large Ovarian Tumor—Operation and Recovery. By HERMAN E. HAYD, M. D. | 37 |
| Nuclear Ophthalmoplegia. By ALVIN A. HUBBELL, M. D. | 39 |
| A Case of Rhinophyma (Rosacea Hypertrophica). By ERNEST WENDE, M. D. | 43 |
| Tubercular Infection in Street Cars. By WILLIAM G. BISSELL, M. D. | 46 |
| Cardiac Embolism Following Abdominal Section. By C. C. FREDERICK, B. S., M. D. | 48 |
| Synchronous Amputations. By CLAYTON M. DANIELS, M. D. | 50 |
| Enteric Fever Complicating Pregnancy. By G. A. HIMMELSBACH, M. D. | 57 |
| Obstructions Within the Upper Respiratory Tract of Children; their Relation to General Health. By HENRY J. MULFORD, M. D. | 60 |
| NEW INVENTIONS. | |
| A Sanitary Burial Casket | 63 |
| SPECIAL ARTICLE. | |
| Fifty Years of Medical Journalism. By WILLIAM WARREN POTTER, M. D. | 65 |
| EDITORIAL. | |
| An Electric Railway Ambulance | 114 |
| Professor Thomas H. Huxley | 119 |
| Obituary | 122 |
| Personal | 124 |
| Society Meetings | 126 |

